# The antifibrotic effect of Vildagliptin and Diaminodiphenyl Sulfone in murine schistosomiasis *mansoni*

**DOI:** 10.1038/s41598-025-91955-4

**Published:** 2025-03-24

**Authors:** Amira S. Hendawy, Abdel-Nasser A. Sabra, Mina Y. George, Eman Rashad, Ebtehal El-Demerdash, Sanaa S. Botros

**Affiliations:** 1 Department of Pharmacology, Theodor Bilharz Research Institute, Warrak El-Hadar, P.O. Box 30, Imbaba, Giza, 12411 Egypt; 2 Department of Pharmacology and Toxicology, Faculty of Pharmacy, Ain Shams University, Abasia, Cairo, 11566 Egypt; 3 Department of Cytology and Histology, Faculty of Veterinary Medicine, Cairo University, Giza, 12211 Egypt

**Keywords:** *Schistosoma mansoni*, Vildagliptin, Diaminodiphenyl sulfone, Dipeptidyl Peptidase IV, Inflammation, Fibrosis, Drug discovery, Gastroenterology

## Abstract

Schistosomiasis drastically affects human health, where *S. mansoni*-induced hepatic fibrosis remains a serious problem with no available drug yet. The current study aimed to evaluate the hepatoprotective effects of Vildagliptin (Vilda), Diaminodiphenyl Sulfone (DDS), and their combination (Vilda/DDS) against *S. mansoni*-induced hepatic fibrosis and elucidate their underlying molecular mechanisms. *S.mansoni*-infected mice were administered praziquantel (PZQ) for two consecutive days, or Vilda, DDS, and Vilda/DDS for 14 consecutive days. Schistosomiasis-induced hepatic fibrosis was assessed parasitologically, biochemically, and pathologically. Results revealed that Vilda, DDS, and Vida/DDS treatments significantly reduced worm count, oogram stages, ova count, and ameliorated the granulomatous inflammatory reactions and hepatotoxicity indices. Moreover, they enhanced hepatic Nrf2/HO-1 pathway with significant increasing SOD and reducing MDA levels. Furthermore, they significantly downregulated the hepatic TLR4/NF-κB and NLRP3 inflammasome pathways leading to a significant reduction in TNF-α and caspase-1 levels which is important in the activation of IL-1β and caspase-3. Notably, significant downregulation in hepatic TGF-β1, α-SMA, and MMP-9 expressions were also recorded. In conclusion, Vilda/DDS showed antioxidant, anti-inflammatory and antifibrotic activities in comparison to either Vilda or DDS alone against *S. mansoni*-induced hepatic fibrosis. Therefore, Vilda/DDS is a promising approach for managing *S. mansoni* infection, liver fibrosis, and associated disease morbidity.

## Introduction

Schistosomiasis is a major worldwide neglected tropical disease that greatly threatens human health by affecting morbidity and mortality^1,2^. Schistosomiasis transmission has been reported in 78 countries, mostly common in poor countries of the Middle East, South America, Southeast Asia, and sub-Saharan Africa, it is estimated to affect more than 250 million people causing approximately 280,000 deaths annually^3^. Through climate change and globalization, schistosome parasites expand their original tropical habitats and are expected to invade areas with rather moderate climates such as Corsica or Almeria (Spain)^4^. Although many countries have taken steps towards managing the disease, most sub-Saharan African countries are still groaning under the burden of the disease^5,6^. In the chemotherapy of schistosomiasis, cure, and lessened disease morbidity are the aim targets, Praziquantel (PZQ) is still the cornerstone in the chemotherapy of the disease^7^. Although the effectiveness of PZQ on hepatic lesions is mainly ascribed to parasite eradication^8^, the reduction in fibrosis appears to depend on the time at which PZQ was provided, specifically before the fibrosis developed^9^. Hepatic fibrosis as a multicellular wound-repairing process is a life-threatening complication, including extreme extracellular matrix component aggregation^10^. Despite chronic HCV, alcohol abuse, and non-alcoholic steatohepatitis are the main causes of liver fibrosis, *Schistosoma mansoni* infection invariably results in liver fibrosis. Hepatic periportal fibrosis is caused by the T cell-dependent granuloma that develops around schistosome eggs. Experimental models of infection have shown that cytokines highly regulate granuloma and fibrosis.

*S. mansoni* induces more than one inflammasome during infection, laying of eggs by *Schistosoma* parasites results in the generation of free radicals, inducing oxidative stress^11^ with decreased levels of the protective antioxidant enzymes; the Nrf2, SOD, and HO-1^12^**.** Moreover, *S. mansoni* activates TLR4^13^ which initiates NLRP3 inflammasomes^14^ and the crosstalk between these signaling pathways^15^, activates caspase-1 and IL-1β^16,17^. *S. mansoni-*induced inflammation results also in the overproduction of pro-inflammatory cytokines (TNF-α and IL-1β)^18,19^. These cytokines and chemokines as TGF-β1 trigger the activation of hepatic stellate cells that increase the inflammatory response and may prompt α-SMA^20^ which also encourages granuloma formation^21^ that progressively transform into fibrous tissues in the periportal areas^1^. Inflammatory liver diseases also demonstrate the link between apoptosis and inflammation in the afflicted liver^22^ where an increased level of caspase 3 was recorded resulting in cellular apoptosis^23^.

Vildagliptin (Vilda) is a dipeptidyl peptidase -4 inhibitor (DPPI-4), an oral antidiabetic drug, a membrane-associated peptidase that is expressed widely in tissues^24^. Vilda declines liver DPP-4 activity, therefore, possessing anti-inflammatory, antioxidant, and anti-fibrotic properties^25–28^. Diaminodiphenyl sulfone (DDS) is an antibacterial and anti-inflammatory drug that has been used since 1950 to treat a variety of conditions, including inflammatory bowel disease, leprosy, and malaria^29–31^, also it is recognized as an anti-oxidative stress effect^32^. This study aims to investigate the efficacy of Vilda and DDS drugs for treating schistosomiasis and provide insights into how treatments affect the pathophysiology of the disease, the underlying molecular mechanisms, and the expected effects on the development of hepatic fibrosis.

## Results

The current study showed that normal mice administered Vilda, DDS, and Vilda/DDS did not induce any significant changes in any of the assessed markers when compared to the normal control group.

### Effect of Vilda, DDS, and Vilda/DDS on the parasitological criteria and histopathological changes.

In *S. mansoni-*infected mice, 30% of invading cercariae developed into worms, with oogram patterns showing different egg developmental stages (immature, mature, and dead). Infected mice treated with Vilda, DDS, Vilda/DDS, and PZQ revealed a significant reduction in worm burden, oogram (immature & mature egg stages), and ova count with a significant increase in dead eggs stages when compared to *S. mansoni-*infected mice. Infected mice treated with PZQ and Vilda/DDS showed a complete disappearance of immature eggs with a significant increase in the number of dead eggs and a decrease in hepatic tissue egg load, the difference between both groups was insignificant (Table 1).

**Table 1:** Effect of Vilda, DDS, Vilda/DDS, and PZQ on worm load, percentage of egg developmental stages, tissue egg load, and granuloma size in *S. mansoni-*infected mice.

Animal groups	Total worms	Percentage of egg developmental stages			Tissue egg load		
		Total immature	Mature	Dead	Hepatic count × 10^3^	Intestinal count × 10^3^	Granuloma Size (μm)
Infected	24.3 ± 1.0	54.5 ± 1.6	49.8 ± 4.3	5.0 ± 0.3	14.5 ± 1.7	24.2 ± 0.8	350.2 ± 12.9
Inf + PZQ (500 mg/kg/day)	2.5 ± 0.2^a^ 89.70%	0.0 ± 0.0^a^ 100%	24.2 ± 1.5^a^ 51.50%	78.5 ± 1.8^a^ 93.60%	5.2 ± 0.4^a^ 64.20%	4.7 ± 0.3^a^ 80.40%	252.9 ± 15.2^a^ 28%
Inf + Vilda/DDS (20 mg/kg) + (50 mg/g)	7.7 ± 0.6^ab^ 68.50%	0.0 ± 0.0^a^ 100%	32.7 ± 2.0^a^ 34.50%	69.2 ± 2.4^a^ 92.80%	7.3 ± 0.4^a^ 50%	8.2 ± 0.3^ab^ 66%	245.0 ± 12.3^a^ 30%
Inf + Vilda (20 mg/kg)	12.7 ± 1.5^abc^ 47.90%	26.5 ± 2.4^abc^ 51.40%	38.0 ± 1.0^ab^ 23.80%	38.5 ± 2.7^abc^ 87%	9.3 ± 0.9^ab^ 35.80%	13.4 ± 1.2^abc^ 44.70%	227.5 ± 8.0^a^ 35%
Inf + DDS (50 mg/kg)	11.0 ± 1.3^ab^ 54.80%	20.0 ± 2.89^abc^ 63.30%	35.8 ± 2.0^ab^ 28.10%	48.3 ± 3.07^abc^ 89.70%	8.4 ± 0.7^a^ 41.90%	12.5 ± 0.7^abc^ 48.50%	292.0 ± 12.^a^ 17%

Inf + PZO, infected mice treated with paraziquantel; Inf + Vilda/DDS, infected mice treated with a combination of vildagliptin and diaminodiphenyl sulphone; Inf + Vilda, infected mice treated with vildagliptin; Inf + DDS, infected mice treated with diaminodiphenyl sulphone. All treatments were administered for 14 consecutive days, except PZQ, which was administered for two consecutive days. Data are presented as mean ± SEM, (n = 6) and analyzed by one-way ANOVA followed by Tukey’s multiple comparisons test, values represent percentage decrease or increase relative to infected control.

^a^Significant difference from the *S. mansoni-*infected group (Infected).

^b^Significant difference from Inf + PZQ.

^c^Significant difference from Inf + Vilda/DDS at P < 0.05.

**Fig. 1:**
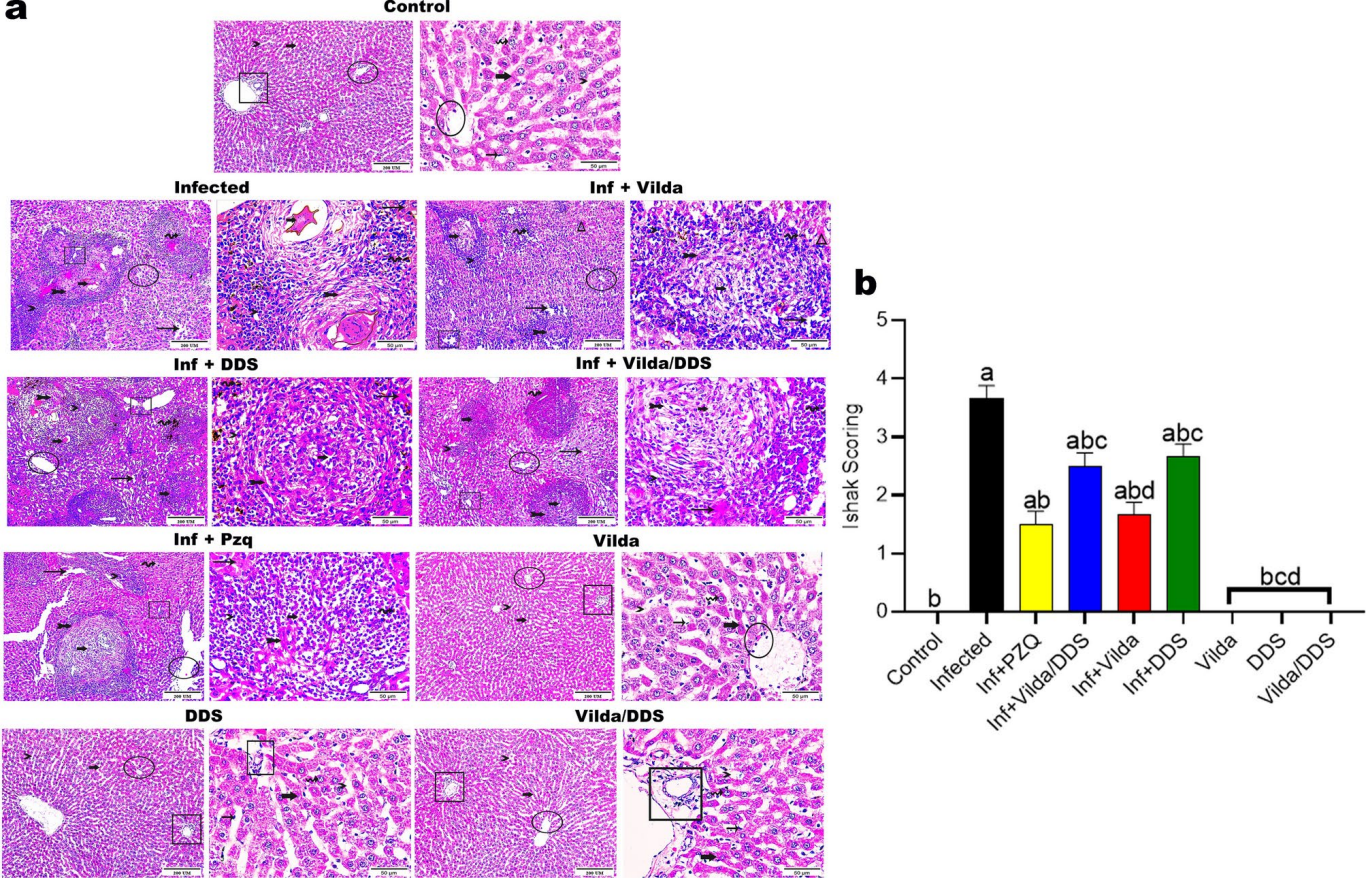
Effect of Vilda, DDS, Vilda/DDS on histopathological alterations along hepatic tissue of *S. mansoni-*infected mice. (**a**) photomicrographs of hepatic sections stained with H&E (× 100, × 400 & scale bar = 200μm, 50μm, respectively) as follows: liver sections of normal control and normal treated groups (Control, Vilda, DDS, and Vilda/DDS) displayed standard hepatic parenchyma along its central vein (circle), portal area (cube), hepatic cords (thick arrow), hepatocyte nucleus (wave arrow), blood sinusoids (arrowheads) as well as Von kupffer cells (thin arrows). Liver sections from (Infected to Inf + PZQ) highlighted the histopathological alterations that assembled as granulomas (thick arrow), inflammatory cells encircled granuloma (arrowhead), macrophage pigment (wave arrow), collagen fibers (double head arrow), inflammatory cells bordered portal area (cube) and central vein (circle), necrotic areas (triangle), as well as vacuolations, dilated and collapsed blood sinusoids (thin arrow). (**b**) Ishak scoring system. Data are presented as mean ± SEM, (n = 6), analyzed by one-way ANOVA followed by Tukey multiple comparisons, ^a^Significantly different from the normal untreated group (Control), ^b^Significantly different from the *S. mansoni* infected untreated group (infected), ^c^Significantly different from Inf + PZQ group, and ^d^Significantly different from Inf + Vilda/DDS group, at P < 0.05. Inf + PZO, infected mice treated with paraziquantel; Inf + Vilda/DDS, infected mice treated with a combination of vildagliptin and diaminodiphenyl sulphone; Inf + Vilda, infected mice treated with vildagliptin; Inf + DDS, infected mice treated with diaminodiphenyl sulphone. All treatments were administered for 14 consecutive days, except PZQ for two consecutive days.

In (Fig. 1a) Histopathological examination of liver sections of normal control and normal treated mice with Vilda, DDS, and Vilda/DDS showed normal hepatic architecture. Moreover, liver sections of *S. mansoni-*infected mice displayed several cellular granulomas homing fresh ova in its center encircled by obvious aggregation of inflammatory cells bordering the portal area and central vein, Hepatic cords exhibited degenerative changes with dilation in blood sinusoids, and highlighting the highest amount of collagen fibers. Conversely, *S. mansoni-*infected mice treated with Vilda, DDS, Vilda/DDS, and PZQ exhibited a significant reduction in granuloma size encircled by a low number of inflammatory cells when compared to *S. mansoni*-infected mice. Granulomas in livers of infected mice treated with Vilda and Vilda/DDS showed the highest reduction in granuloma size, with a decrease in the number of intact granulomas and an increase in the number of degenerated ones (67% & 57%) respectively, the difference in granuloma size was insignificant when compared to PZQ treated group.

Besides, Ishak scoring system for assessment of liver tissue fibrosis, revealed that normal control and normal treated mice with Vilda, DDS, and Vilda/DDS didn’t show significant differences between them. On the other hand, *S. mansoni*-infected mice showed the highest fibrotic score. Treatment of infected mice with Vilda, DDS, Vilda/DDS, and PZQ showed a significant reduction in the scoring by 55%, 27%, 32% and 59%, respectively, when compared to *S. mansoni*-infected mice (Fig. 1b).

**Fig. 2:**
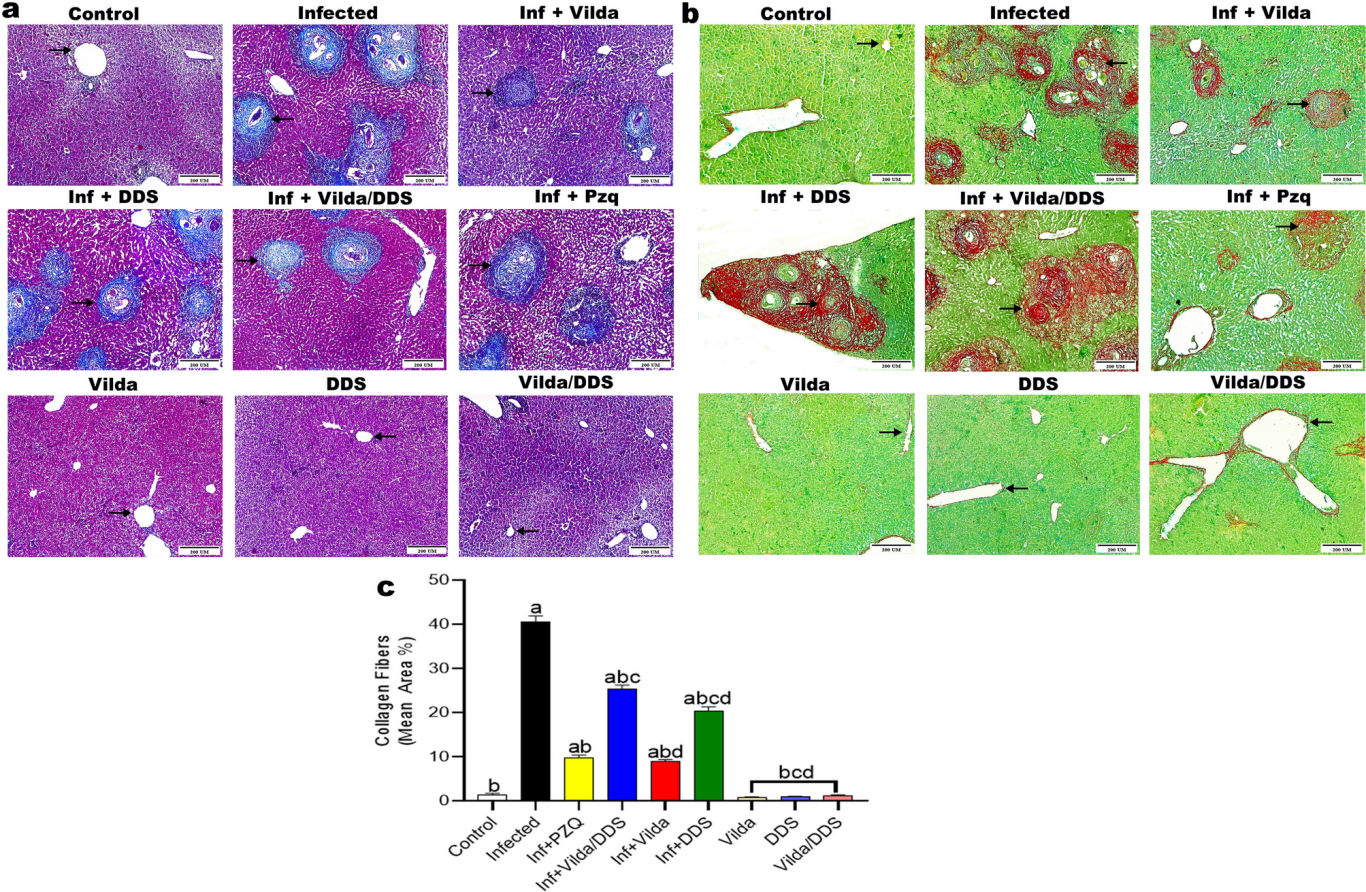
Effect of Vilda, DDS, and Vilda/DDS on collagen fibers along hepatic tissues of *S. mansoni-*infected mice. Representative photomicrographs of hepatic sections stained with (**a**) Masson trichrome stain, and (**b**) Picrosirius red- fast green stain, (× 100, scale bar = 200μm) as follows: liver sections of normal control and normal treated groups (Control, Vilda, DDS, and Vilda/DDS) marked scarce amount of collagen fibers (arrows), the infected group highlighted the highest amount (arrow), Inf + Vilda and Inf + PZQ emphasized few amounts (arrows), and Inf + DDS and Inf + Vilda/DDS displayed moderate low and moderate high amounts of collagen fibers, respectively (arrows), (red areas): collagenous fibers, (green areas): non-collagenous protein. (**c**) quantitative analysis of collagen fiber. Data are presented as mean ± SEM, (n = 6), analyzed by one-way ANOVA followed by Tukey multiple comparisons, ^a^Significantly different from the normal untreated group (Control), ^b^Significantly different from the *S. mansoni-*infected untreated group (infected), ^c^Significantly different from Inf + PZQ group, and ^d^Significantly different from Inf + Vilda/DDS group, at P < 0.05. Inf + PZO, infected mice treated with paraziquantel; Inf + Vilda/DDS, infected mice treated with a combination of vildagliptin and diaminodiphenyl sulphone; Inf + Vilda, infected mice treated with vildagliptin; Inf + DDS, infected mice treated with diaminodiphenyl sulphone. All treatments were administered for 14 consecutive days, except PZQ for two consecutive days.

Furthermore, histochemical findings highlighted the consequence of investigated drugs on collagen deposition in liver tissues using Masson Trichrome Stain (Fig. 2a) and Sirus Red Stain (Fig. 2b). Results revealed that liver sections of normal control and normal mice treated with Vilda, DDS, and Vilda/DDS marked scarce amount of collagen fibers along central vein and portal areas without significant differences between them, but with significant differences from other studied groups. *S. mansoni*-infected mice highlighted the highest amount of collagen fibers encircling ova of Schistosoma constructing granuloma with significant differences from other studied groups. On the other hand, *S. mansoni*-infected mice treated with Vilda, DDS, Vilda/DDS, and PZQ exhibited a significant reduction in the amount of collagen fibers by 78%, 50%, 38%, and 76%, respectively (Fig. 2c), as compared to *S. mansoni*-infected mice. In brief, infected mice treated with either Vilda or PZQ emphasized few amounts of collagen fibers encircling granuloma without significant difference between them but with significant difference from other studied groups. Infected mice treated with either DDS or Vilda/DDS displayed moderate low or moderate high amounts of collagen fibers encircling granuloma, respectively, with significant differences between them and other studied groups (Fig. 2).

### Effect of Vilda, DDS, and Vilda/DDS on hepatotoxicity markers.

Regarding hepatic biomarkers expressing hepatocyte integrity (ALT, AST, Albumin, and liver index), we reported that *S. mansoni*-infected mice showed a significant increase in ALT, AST, and liver index with a significant decrease in serum albumin levels when compared to normal control mice. Whereas, *S. mansoni*-infected mice treated with Vilda, DDS, Vilda/DDS, and PZQ showed a significant reduction in ALT, AST, and liver index with a significant increase in albumin level when compared to *S. mansoni*-infected mice (Table 2). Infected mice treated with Vilda/DDS and PZQ did not reveal any significant differences between each other or when compared to normal control group in liver enzyme levels (ALT, AST, and albumin).

**Table 2:** Effect of Vilda, DDS, and Vilda/DDS on liver biomarkers of *S. mansoni-*infected mice.

Animal groups	ALT (U/mL)	AST (U/mL)	Albumin (g/dL)	Liver Index (%)
Control	64.7 ± 5.5^b^	86.0 ± 1.5^b^	3.23 ± 0.05^b^	6.9 ± 0.4^b^
Infected	119.3 ± 2.5^a^	144.1 ± 1.7^a^	2.1 ± 0.1^a^	10.8 ± 0.4^a^
Inf + PZQ (500 mg/kg/day)	72.7 ± 5.7^b^ 39%	96.1 ± 1.5^b^ 33%	3.1 ± 0.1^b^ 31.2%	8.0 ± 0.3^b^ 26%
Inf + Vilda/DDS (20 mg/kg) + (50 mg/kg)	76.1 ± 3.4^b^ 36%	96.3 ± 1.8^b^ 33%	3.1 ± 0.1^b^ 31.5%	8.8 ± 0.3^ab^ 19%
Inf + Vilda (20 mg/kg)	86.7 ± 4.9^ab^ 27%	100.2 ± 4.4^ab^ 30%	2.9 ± 0.0^ab^ 25.3%	9.2 ± 0.2^ab^ 15%
Inf + DDS (50 mg/kg)	87.9 ± 4.5^ab^ 26%	104.1 ± 3.3^ab^ 28%	2.9 ± 0.1^ab^ 24.8%	9.4 ± 0.2^a^ 13%
Vilda (20 mg/kg)	66 ± 1.4^b^	87.9 ± 1.9^b^	3.29 ± 0.09^b^	5.5 ± 0.3^bcd^
DDS (50 mg/kg)	67.2 ± 0.4^b^	86.8 ± 2.3^b^	3.22 ± 0.12^b^	6.6 ± 0.4^bd^
Vilda/DDS (20 mg/kg + 50 mg/kg	66 ± 0.8^b^	86.2 ± 2.5^b^	3.30 ± 0.07^b^	5.8 ± 0.2^bcd^

ALT, Alanine aminotransferase; AST, Aspartate aminotransferase; Inf + PZO, infected mice treated with paraziquantel; Inf + Vilda/DDS, infected mice treated with a combination of vildagliptin and diaminodiphenyl sulphone; Inf + Vilda, infected mice treated with vildagliptin; Inf + DDS, infected mice treated with diaminodiphenyl sulphone. Data are presented as mean ± SEM, (n = 6), and analyzed by one-way ANOVA followed by Tukey multiple comparisons, values represent percentage decrease or increase relative to infected control.

^a^Significantly different from normal untreated group (Control).

^b^Significantly different from *S. mansoni-*infected untreated group (Infected).

^c^Significant difference from Inf + PZQ.

^d^Significant difference from Inf + Vilda/DDS at P < 0.05. All treatments were administered for 14 consecutive days, except PZQ, which was administered for two consecutive days.

### Effect of Vilda, DDS, and Vilda/DDS on oxidative stress markers

**Fig. 3:**
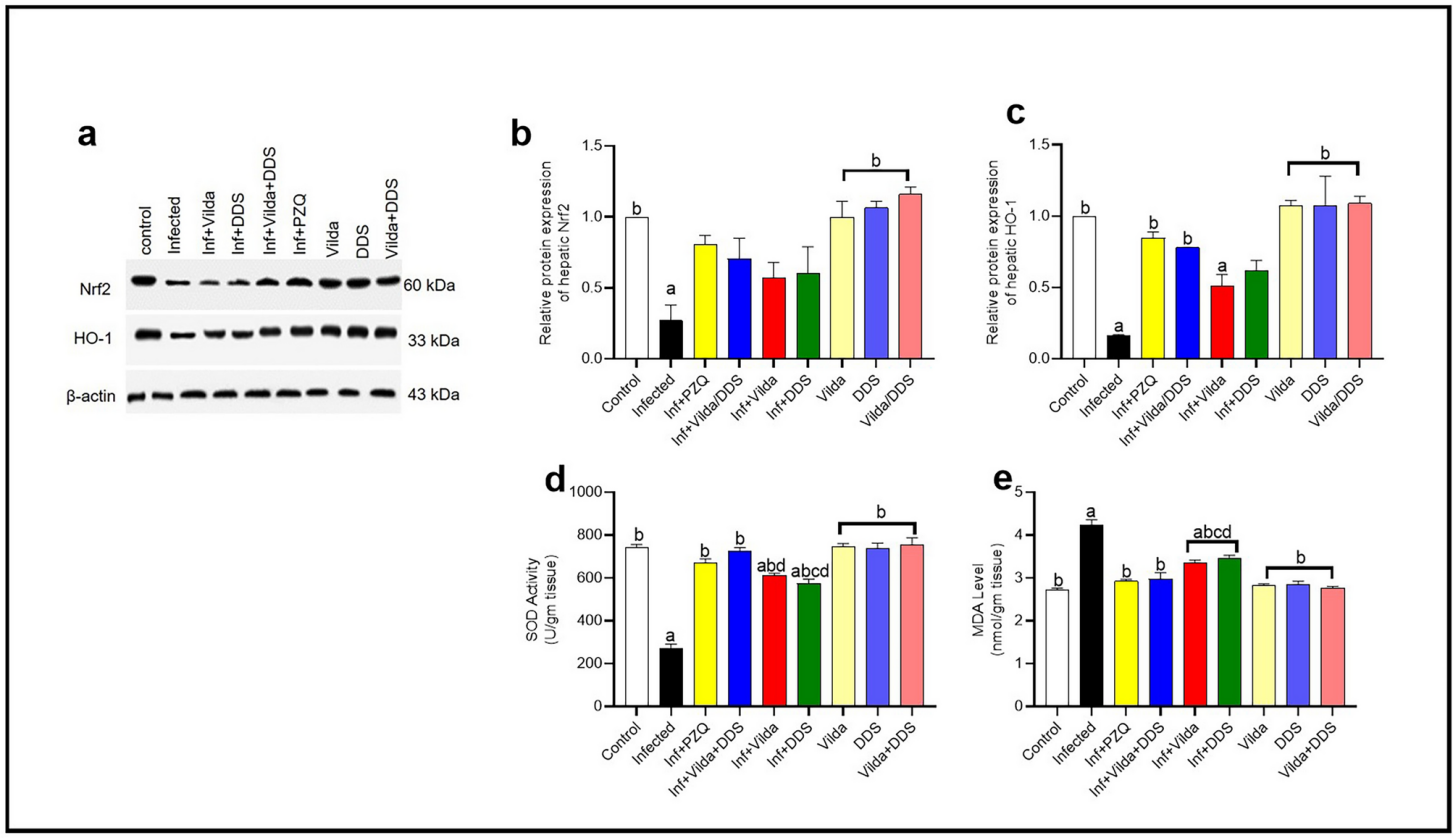
Effect of Vilda, DDS, and Vilda/DDS on oxidative stress markers of *S. mansoni-*infected mice. (**a**) Representative western blot images for Nrf2 and HO-1 protein expression. Blot densitometry quantitation using Image J analysis software for (**b**) nuclear factor erythroid 2–related factor 2 (Nrf2) and (**c**) heme oxygenase-1 (HO-1) (n = 2). Two full-length blots are included in the Supplementary file. (**d**) Representative Hepatic level of superoxide dismutase (SOD) and (**e**) malondialdehyde (MDA), data are presented as mean ± SEM (n = 6). Data analysis was done using one-way ANOVA followed by Tukey multiple comparisons. ^a^Significantly different from the normal untreated group (Control), ^b^Significantly different from the *S. mansoni*-infected untreated group (infected), ^c^Significantly different from Inf + PZQ group, and ^d^Significantly different from Inf + Vilda/DDS group, at P < 0.05. Inf + PZO, infected mice treated with paraziquantel; Inf + Vilda/DDS, infected mice treated with a combination of vildagliptin and diaminodiphenyl sulphone; Inf + Vilda, infected mice treated with vildagliptin; Inf + DDS, infected mice treated with diaminodiphenyl sulphone. All treatments were administered for 14 consecutive days, except PZQ for two consecutive days.

*S. mansoni*-infected mice showed a significant increase in the oxidative stress markers, as revealed by a significant increase of MDA levels concomitant with a significant decrease in Nrf2 and HO-1 expressions as well as SOD activity as compared to the control group. Infected mice treated with Vilda, DDS, Vilda/DDS, and PZQ showed pronounced elevation in liver expression of Nrf2 by 52%, 55%, 61% and 66%, respectively as compared to *S. mansoni*-infected mice (Fig. 3a,b). Furthermore, liver expression of HO-1 was markedly elevated in infected mice treated with Vilda and DDS by 68% and 73%, respectively as compared to *S. mansoni*-infected mice (Fig. 3a,c). Treatment of the infected mice treated with either the combination Vilda/DDS or the standard therapy, PZQ showed a significant elevation of HO-1 expression by 79%, and 81%, respectively as compared to *S. mansoni*-infected mice (Fig. 3a,c). Additionally, infected mice treated with Vilda, DDS, Vilda/DDS, and PZQ showed a significant increase in SOD activity by 56%, 53%, 62%, and 59%, respectively (Fig. 3d) along with, a significant decrease in MDA levels by 21%, 18%, 31%, and 31.2%, respectively (Fig. 3e) as compared to *S. mansoni*-infected mice. Noticeably, infected mice treated with either the combination of Vilda/DDS or PZQ did not reveal significant differences between each other or when compared to the normal control group resulting in normalization of Nrf2, HO-1, SOD, and MDA levels.

### Effect of Vilda, DDS, Vilda/DDS on inflammatory and apoptotic markers

*S. mansoni*-infected mice showed a significant increase in the inflammatory and apoptotic markers as demonstrated by significant increase in the hepatic expression of TLR4, NLRP3, caspase-1, and NF-κB p65 along with significant increase in TNF-α and IL-1β levels, as well as, significant elevation in the apoptotic marker (caspase-3), when compared to the control group.

**Fig. 4:**
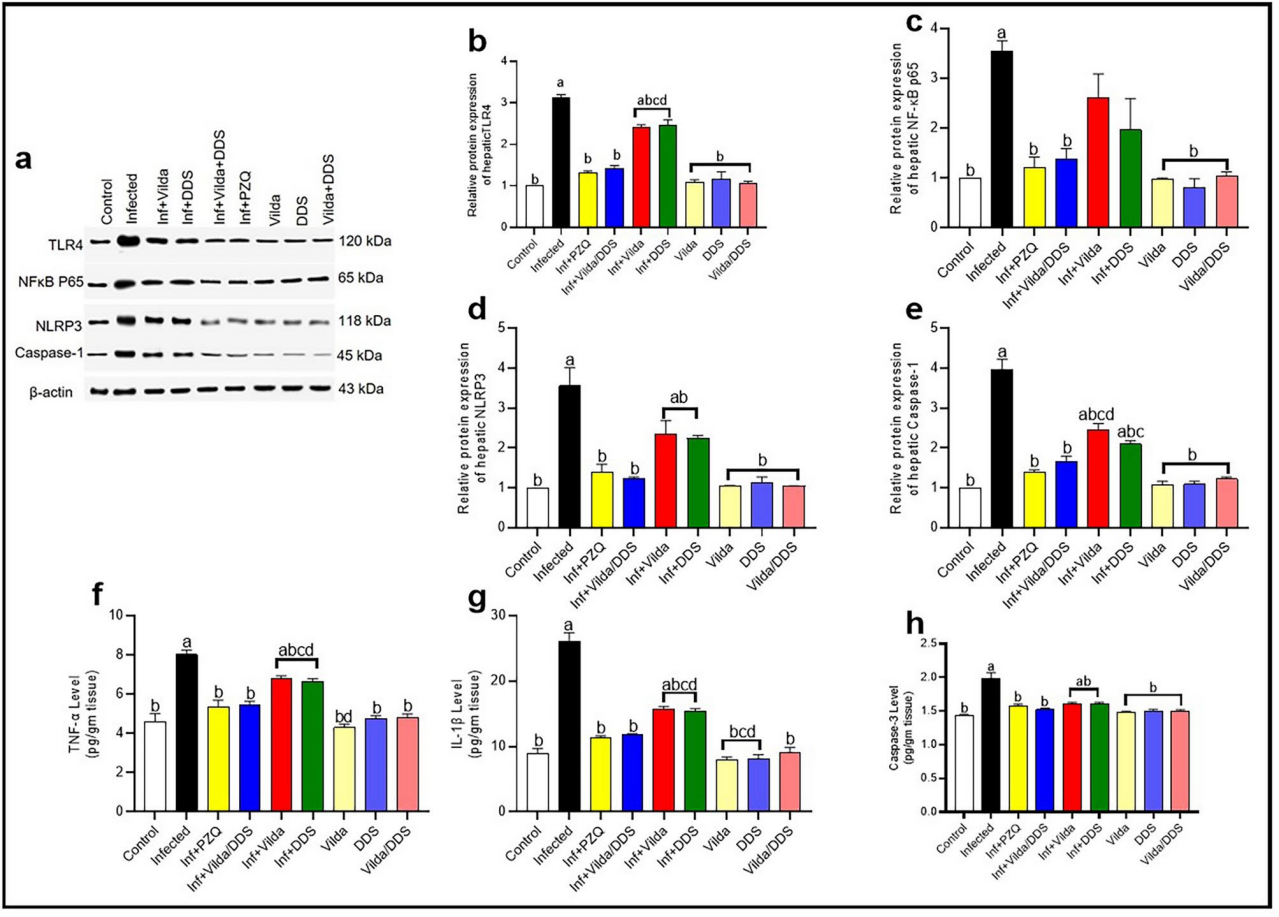
Effect of Vilda, DDS, Vilda/DDS on inflammatory and apoptotic markers in *S. mansoni-*infected mice. (**a**) Representative western blot images for TLR4, NF-κB p65, NLRP3 and Caspase-1 protein expression. Blot densitometry quantitation using Image J analysis software for (**b**) toll-like receptor 4 (TLR4), (**c**) nuclear factor kappa-B p65 (NF-κB p65), (**d**) NOD-like receptor proteins (NLRP3), and (**e**) Caspase-1 (n = 2). Two full-length blots are included in the Supplementary file. (**f**) Representative hepatic level of tumor necrosis factor-alpha (TNF-α), (**g**) interleukin-1 beta (IL-1β), and (**h**) Caspase-3 as an apoptotic marker, data are presented as mean ± SEM (n = 6). Data analysis was done using one-way ANOVA followed by Tukey multiple comparisons. ^a^Significantly different from the normal untreated group (Control), ^b^Significantly different from the *S. mansoni*-infected untreated group (infected), ^c^Significantly different from Inf + PZQ group, and ^d^Significantly different from Inf + Vilda/DDS group, at P < 0.05. Inf + PZO, infected mice treated with paraziquantel; Inf + Vilda/DDS, infected mice treated with a combination of vildagliptin and diaminodiphenyl sulphone; Inf + Vilda, infected mice treated with vildagliptin; Inf + DDS, infected mice treated with diaminodiphenyl sulphone. All treatments were administered for 14 consecutive days, except PZQ for two consecutive days.

*S. mansoni-*infected mice treated with Vilda, DDS, Vilda/DDS, and PZQ revealed a marked reduction in the hepatic expression of NF-κB p65 by 27%, 45%, 61% and 66%, respectively (Fig. 4a,c), associating with significant reduction in the hepatic expression of TLR4 by 22%, 21%, 54% and 58%, respectively (Fig. 4a,b), NLRP3 by 34%, 37%, 66% and 61%, respectively (Fig. 4a,d), and Caspase-1 by 38%, 47%, 58% and 65%, respectively (Fig. 4a,e) as compared to as compared to *S. mansoni*-infected mice. Further, *S. mansoni-*infected mice treated with Vilda, DDS, Vilda/DDS, and PZQ showed significant decrease in the inflammatory cytokines TNF-α by15%, 17%, 32% and 33%, respectively (Fig. 4f) and IL-1β by 39%, 41%, 55% and 57%, respectively (Fig. 4g), as well as a significant reduction in the activity of caspase-3 by 19%, 19%, 24% and 21% respectively (Fig. 4h) as compared to *S. mansoni-*infected mice.

Noticeably, infected mice treated with Vilda/DDS and PZQ did not show any significant differences in comparison to each other or to the normal control group resulting in normalization of the above mentioned inflammatory and apoptotic markers.

### Effect of Vilda, DDS, and Vilda/DDS on fibrotic markers

Liver sections from normal control mice and normal mice treated with different treatment regimens revealed comparable scarce hepatocytes with positive cytoplasmic reactivity to TGF-β1 and α-SMA antibodies. However, liver sections from *S. mansoni-*infected mice showed pronounced positive expression for TGF-β1 and α-SMA within ova of *S. mansoni,* fibroblast encircling granuloma, inflammatory cells with nuclear expression, and neighboring with cytoplasmic reactivity when compared to their corresponding normal control group.

**Fig. 5:**
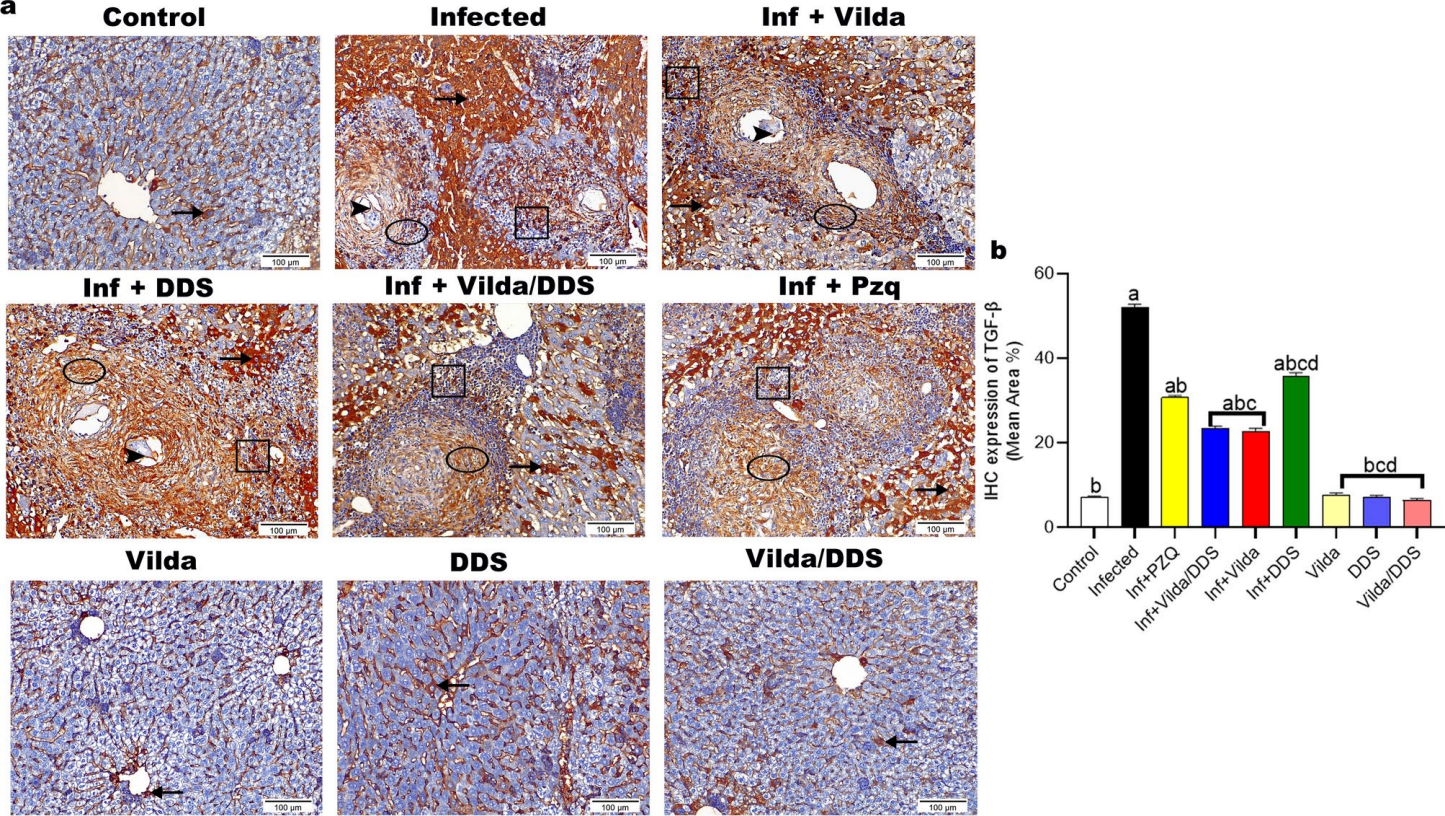
Effect of Vilda, DDS, and Vilda/DDS on TGF-β as a fibrotic marker on *S. mansoni-*infected mice. (**a**) Representative photomicrographs of immunohistochemical staining (IHC) of hepatic sections with transforming growth factor beta (TGF-β) (× 200, scale bar = 100µm) showing as follows: liver sections of normal control and normal treated groups (Control, Vilda, DDS, and Vilda/DDS) demonstrated scarce hepatocytes with positive cytoplasmic reactivity to TGF-β antibody (arrow). Liver sections from (Infected to Inf + PZQ) revealed positive expression of TGFβ along ova of *Schistosoma* (arrowhead), fibroblast encircling granuloma (circle), inflammatory cells with nuclear expression (cube), and neighboring hepatocytes with cytoplasmic reactivity (arrow). The expressions were graded as intense in the Infected group, high in Inf + DDS group, moderate in Inf + PZQ**,** and few in Inf + Vilda and Inf + Vilda/DDS groups. (**b**) Quantitative analysis of the IHC expression of hepatic TGF-β. Data are presented as mean ± SEM, (n = 6) and analyzed by one-way ANOVA followed by Tukey multiple comparisons. ^a^Significantly different from normal control group (Control), ^b^Significantly different from the infected control group (infected), ^c^Significantly different from Inf + PZQ group, and ^d^Significantly different from Inf + Vilda/DDS group, at P < 0.05. Inf + PZO, infected mice treated with paraziquantel; Inf + Vilda/DDS, infected mice treated with a combination of vildagliptin and diaminodiphenyl sulphone; Inf + Vilda, infected mice treated with vildagliptin; Inf + DDS, infected mice treated with diaminodiphenyl sulphone. All treatments were administered for 14 consecutive days, except PZQ for two consecutive days.

**Fig. 6:**
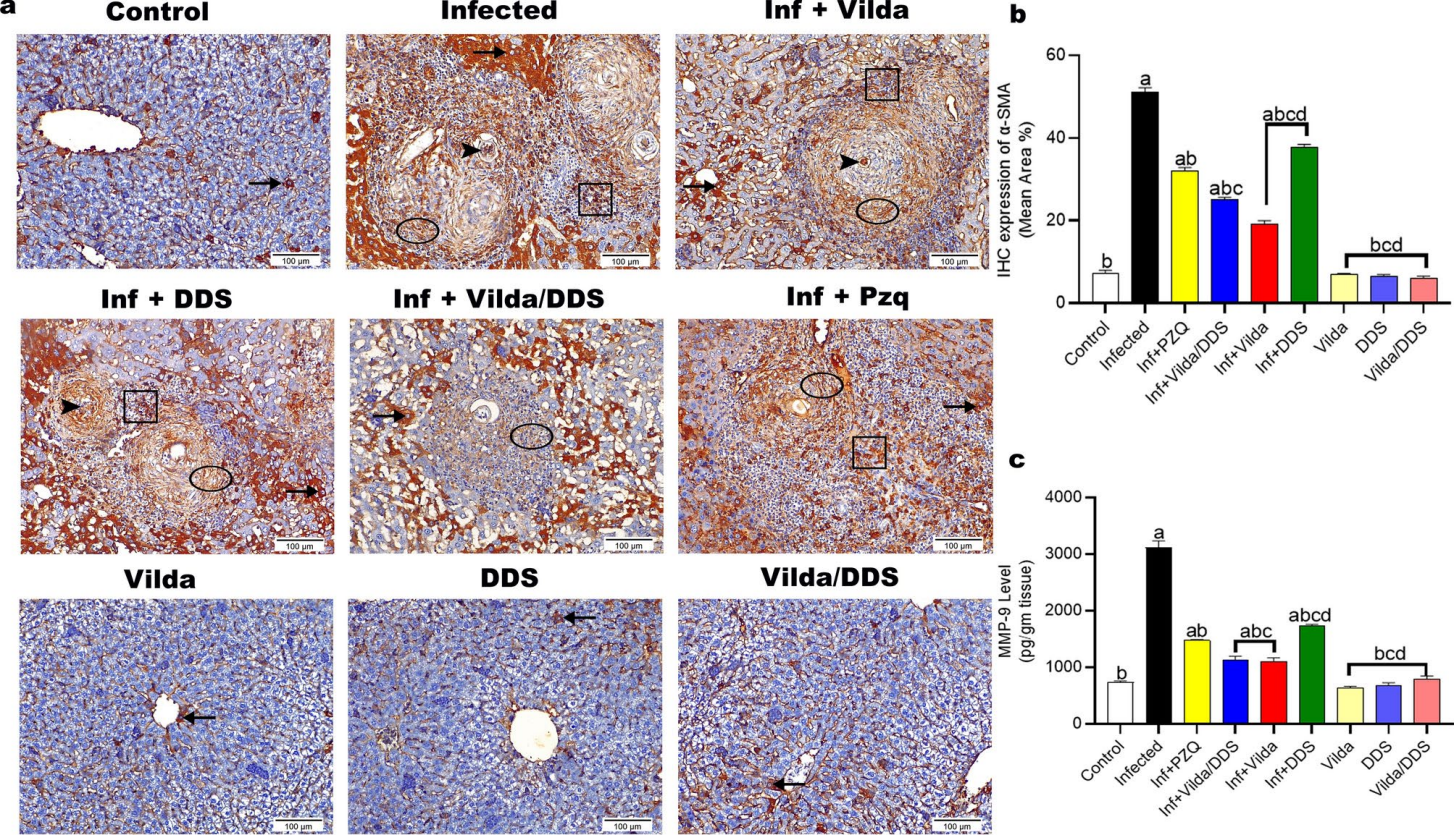
Effect of Vilda, DDS, and Vilda/DDS on α-SMA and MMP-9 as fibrotic markers on *S. mansoni-*infected mice. (**a**) Photomicrographs of immunohistochemical staining (IHC) of hepatic tissues with alpha-smooth muscle actin (α-SMA) (× 200, scale bar = 100µm) as follows: liver sections of normal control and normal treated groups (Control, Vilda, DDS, and Vilda/DDS) marked scarce hepatocytes with positive cytoplasmic reactivity to α-SMA antibody (arrow). Liver sections from (Infected to Inf + PZQ) highlighted positive expression of α-SMA along ova of *Schistosoma* (arrowhead), fibroblast encircling granuloma (circle), inflammatory cells with nuclear expression (cube), and surrounding hepatocytes with cytoplasmic reactivity (arrow). The expressions were graded as intense in the Infected group, high in Inf + DDS group**,** moderately high in Inf + PZQ group**,** moderately low in Inf + Vilda/DDS group**,** and few in Inf + Vilda group. (**b**) Quantitative analysis of hepatic α-SMA IHC expression; **(c)** Hepatic level of matrix metallopeptidase-9 (MMP-9) as a fibrotic marker**.** Data are presented as mean ± SEM, (n = 6) and analyzed by one-way ANOVA followed by Tukey multiple comparisons. ^a^Significantly different from the normal untreated group (Control), ^b^Significantly different from the *S. mansoni*-infected untreated group (infected), ^c^Significantly different from Inf + PZQ group, and ^d^Significantly different from Inf + Vilda/DDS group, at P < 0.05. Inf + PZO, infected mice treated with paraziquantel; Inf + Vilda/DDS, infected mice treated with a combination of vildagliptin and diaminodiphenyl sulphone; Inf + Vilda, infected mice treated with vildagliptin; Inf + DDS, infected mice treated with diaminodiphenyl sulphone. All treatments were administered for 14 consecutive days, except PZQ for two consecutive days.

*S. mansoni-*infected mice treated with Vilda, DDS, Vilda/DDS, and PZQ showed a significant reduction in the expression level of TGF-β1 by 56%, 31%, 55%, and 41%, respectively (Fig. 5a,b), and α-SMA by 62%, 26%, 51%, and 37%, respectively (Fig. 6a,b), when compared to *S. mansoni-*infected group. Hepatic MMP-9 level showed a significant increase in *S. mansoni-*infected mice when compared to normal control group, whereas *S. mansoni-*infected mice treated with Vilda, DDS, Vilda/DDS, and PZQ revealed a significant reduction (65%, 44%, 64%, and 52%) respectively, when compared to *S. mansoni-*infected group (Fig. 6c). Moreover, *S. mansoni-*infected mice treated with Vilda and Vilda/DDS groups were comparable in hepatic fibrosis markers as TGF-β, α-SMA, and MMP-9 and showed higher significant reduction when compared to *S. mansoni-*infected mice treated with PZQ**.**

## Discussion

Up till now, there is no vaccination available for schistosomiasis, leaving the world with only one drug PZQ with some limitations to treat schistosomiasis^33,34^. Even though PZQ cannot resolve liver damage which is why it is still challenging to treat schistosomiasis-related hepatic fibrosis^35,36^. The WHO set aims to eradicate schistosomiasis by 2030 and proposed repurposing of drugs as a possible strategy to manage schistosomiasis infection^37,38^.

Drug repurposing as an alternative approach in drug development, is highly efficient, time-saving, and cost-effective, carries a low risk of failure compared to traditional drug discovery methods and holds great promise, particularly in addressing neglected diseases or diseases of the poor^39,40^. Investigating the potential additional therapeutic benefits of Vilda and DDS the anti-inflammatory and antioxidant drugs when taken alone or in combination, are anticipated to contribute to normalizing and ameliorating the biochemical and histological consequences of schistosomiasis infection.

In the current study, despite neither Vilda nor DDS being anti-schistosomal drugs, their effects alone or in combination as well as PZQ (as positive control) on *S. mansoni-*infected mice, showed a significant reduction in worm burden, the intestinal and hepatic egg load, and in the various stages of egg development (oogram) associated with a significant elevation in dead eggs when compared to *S. mansoni-*infected mice. Lago et al.^41^, stated that Mefenamic acid, a non-steroidal anti-inflammatory medication, has demonstrated anti-schistosomal potential as seen by significantly decreased worm load and egg production. Similarly, *S. mansoni-*infected mice treated with Vilda, DDS, and Vilda/DDS resulted in an improvement in histopathological changes as revealed by a recognizable decrease in the inflammatory granulomatous reaction, which led to a significant reduction in granulomas size, number of ova, and intact granulomas, associated with increased numbers of degenerated granulomas when compared to *S. mansoni-*infected mice. Moreover, *S. mansoni*-infected mice treated with the Vilda/DDS combination showed shrunken and emaciated worm status associated with a significant decrease in the number of worm couples (1.33 ± 0.49 Vs 9.33 ± 0.42), as well as, showed a significant reduction in liver granuloma formation when compared to *S. mansoni*-infected mice. This finding is consistent with the findings of Mostafa et al.^42^, who reported that ginger had varied degrees of anti-schistosomal effects, either by speeding up the clearance of schistosome worms or by decreasing the size of liver granuloma due to *S. mansoni* eggs deposition in liver tissues. Remarkably, *S. mansoni-*infected mice treated with the Vilda/DDS combination and those treated with PZQ were comparable in granuloma size, hepatic tissue egg count, and dead egg stage in the oogram profile with complete disappearance of the immature egg stage. This finding is matched with Pellegrino et al.^43^, who stated that an anti-schistosomal treatment is effective if there is a complete absence of immature stages, a decrease in mature egg stages, and a rise in the number of dead egg stages. Consequently, the Vilda/DDS combination appears to be a potential candidate for schistosomiasis control.

At the same time, this study found that *S. mansoni* infection caused a significant elevation in the serum levels of hepatotoxicity biomarkers (ALT, AST, and liver index) with a significant reduction in the albumin levels when compared to the normal control mice. Such an increase in serum levels may be related to the irritation of the hepatocytes by the released toxins and metabolic products of adult worms, as well as the entrapped schistosome eggs in the liver tissues^44–46^. Meanwhile, treatment of infected mice with Vilda, DDS, Vilda/DDS, and PZQ showed a significant decrease in ALT, AST, and liver index associated with a significant increase in albumin levels.

*S. mansoni* infection was reported to induce an imbalance in the antioxidant/oxidant status, expressed by the suppression in the Nrf2 signaling pathway associated with a significant increase in ROS especially MDA levels, which are expected to end up in the exacerbation of liver fibrosis by stimulating of HSCs and collagen gene transcription^47–49^. The Nrf2/ARE pathway regulates genes involved in many antioxidant systems, which plays a crucial role in the control of cellular redox homeostasis, including the expression of the antioxidant “HO-1 and SOD activity^50^.

Likewise, our findings revealed that *S. mansoni* infection results in an impairment of antioxidant/oxidant defense system as expressed by significant downregulation in Nrf2 and HO-1 hepatic protein expressions with significant reduction in SOD activity that led to significant increase in lipid peroxidation expressed as MDA in comparison to the normal control group. Though, treatment of *S. mansoni-*infected mice with Vilda, DDS, and Vilda/DDS increased the anti-oxidant activity as demonstrated by a prominent elevation in Nrf2 that led to increase in HO-1 hepatic protein expression, resulted in significant increase in SOD activity, with significant reduction in hepatic MDA levels. Our results agree with Ávila et al.^51^ and Diaz-Ruiz et al.^52^, who reported that Vilda and DDS ameliorate oxidative stress markers. Notably, *S. mansoni-*infected mice treated with Vilda/DDS were comparable to those treated with PZQ restoring the antioxidant defense system and liver enzymes. Our results agree with the findings of Hendawy et al.^27^, who stated that hepatoprotective properties of Vilda can be attributed to its antioxidant effects (empowered by an increase in the catalase activity concomitant with a reduction in the MDA level).

*S. mansoni-*infected mice demonstrated potentially enhanced the activity of the TLR/NF-κB and NF-κB/NLRP3 inflammatory pathways, these results, align with Charan et al. and Chen et al.^53,54^, who revealed that *S. mansoni* activates TLR4^13^, which in turn stimulates the transcription factor NF-κB leads to increase in the production of inflammatory genes, including the pro-inflammatory cytokines TNF-α^55,56^. NF-κB activation increased NLRP3 inflammasome expression leading to the activation of caspase-1, subsequently, converts the precursor form of IL-1β into its mature form. In the current study, *S. mansoni-*infected mice treated with Vilda, DDS, and Vilda/DDS revealed a noticeable reduction in NF-κB p65 associating with significant reduction in all other the above-mentioned inflammatory transcription factors and consequently, the inflammatory cytokines. This finding is matched with Hendawy et al.^27^, who reported that Vilda alleviates liver fibrosis in NASH diabetic rats via modulation of oxidative stress and inflammatory cascades. Moreover, Lee et al.^30^, showed that DDS had anti-inflammatory and anti-oxidant properties and reduced the production of inflammatory cytokines and chemokines, including IL-1β, through NF-κB signaling and NLRP3 expression. Noticeably, *S. mansoni-*infected mice treated with Vilda/DDS and PZQ were comparable and showed normalization of hepatic expression of NF-κB p65, Tlr4, NLRP3, and caspase-1, as well as TNF-α, and IL-1β levels.

In this study, *S. mansoni*-infected mice showed an increase in cell injury and apoptotic caspase-3 biomarkers, this finding matches with Ghezellou et al.^57^, who demonstrated that soluble egg antigens of *Schistosoma* encourage immunocyte apoptosis. In addition, *S. mansoni* infection triggered both the intrinsic and extrinsic apoptosis pathways in mouse livers^58,59^. In the current study, *S. mansoni*-infected mice treated with Vilda, DDS, and Vilda/ DDS exhibited a significant reduction in their hepatic apoptotic Caspase-3 level, and this finding agrees with Sherif et al.^60^, who reported that Vildagliptin reduces apoptosis by downregulating hepatic protein expression of the apoptotic marker caspase-3.

Our results revealed that *S. mansoni* infection showed the largest granuloma size around liver tissue-trapped *S. mansoni* eggs with significant elevation in fibrotic marker, TGF-β, α-SMA, and MMP-9. This finding is matched with Fabregat et al.^61^ and Silva et al.^62^, who reported *S. mansoni* infection resulted in liver granuloma and abundant elevation in fibrotic markers. Additionally, *S. mansoni*-infected mice treated with Vilda, DDS, and Vilda/DDS recorded a significant reduction in above mentioned fibrotic markers. Our results are in agreement with Lam et al.^44^, who showed that the antimalarial Casticin had antitumor, anti-inflammatory, and immunomodulatory effects, reduced worm burden, and fibrosis markers as collagen α I, TGF-β, α-SMA on *S. mansoni*-infected mice.

It is worth mentioning that, *S. mansoni*-infected mice treated with the Vilda/DDS combination revealed a higher significant reduction in fibrosis markers when compared to infected mice treated with PZQ, this may point to a more favorable response more than PZQ treatment concerning diminishing periportal fibrosis as a result of S. *mansoni* infection.

In conclusion, the current study highlighted that treatment with Vilda/DDS against Schistosoma *mansoni*-infected mice reduced the parasitological criteria (worm burden, ova count, and oogram stages) and improved histopathological derangements including liver granulomas size. In addition, Vilda/DDS activated the Nrf2-mediated antioxidant response, upregulated key factors as HO-1 expression and SOD activity, ultimately reduced lipid peroxidation and cellular damage caused by the *S. mansoni*-induced oxidative stress. Moreover, it modulated the inflammatory TLR4/NF-κB and NLRP3 signaling pathways, and it is an inflammatory mediator of TNF-α and IL-1β and attenuated hepatic apoptotic marker Caspase 3. Furthermore, Vilda/DDS modulated liver fibrosis through reduction of hepatic stellate cell activation through the inhibition of TGF-β, α-SMA, and MMP-9. This multifaceted mechanism helps to provide protective anti-oxidant, anti-inflammatory, anti-apoptotic, and anti-fibrotic properties, by targeting multiple key nodes in these pathways. Thereby, offering a promising approach for the management of *S. mansoni* infection and the related liver fibrosis and disease morbidity.

## Materials and methods

### Animals

Fifty-four Swiss male albino mice (CD1), weighing 22–25 g were obtained by the Schistosome Biology Supply Center (SBSC), Theodor Bilharz Research Institute, Giza, Egypt (TBRI). Mice were maintained on a standard commercial diet in environmentally controlled conditions (temperature 25 °C; 50% humidity) with free access to water, a 12/12-h automatically timed light/dark cycle were left for one week to acclimatize before *Schistosoma mansoni* infection.

### Ethics approval

The experimental procedures involving animals and their care were conducted in accordance with the ARRIVE guidelines and the Guide for Care and Use of Laboratory Animals published by the US National Institutes of Health (NIH Publication No. 85–23, revised 2011) and was approved by Theodor Bilharz Research Institute, Giza, Egypt (TBRI) ethics committee (PT: 676, 28/2/22).

### Induction of schistosomiasis

Animals were infected via the body immersion technique by the Egyptian strain of *S. mansoni*^63^ with 80 ± 10 *S. mansoni* cercariae, which was obtained by SBSC, TBRI (Giza, Egypt)**.**

### Experimental design

Animals were randomly allocated into nine groups, each of six animals. **Group I (Control)**: normal mice received only the vehicle. **Group II (Infected):** received no treatment and served as *S. mansoni-*infected mice with 80 ± 10 *S. mansoni* cercariae. **Group III (Inf + Vilda):** infected mice treated with Vilda 20 mg/kg^27^ was provided by AUG Pharma Pharmaceutical Company (Cairo, Egypt). **Group IV (Inf + DDS)**: infected mice treated with DDS 50 mg/kg^64^ was purchased from El-Nile Co. for Pharmaceuticals and Chemical Industries (Amreya, Cairo, Egypt). **Group V (Inf + Vilda/DDS):** infected mice treated with a combination of Vilda (20 mg/kg) and DDS (50 mg/kg). **Group VI (Inf + PZQ):** PZQ 500 mg/kg^65^, served as a positive control was provided by EPICO (El-Asher-Men-Ramadan City, Egypt). Parallel Groups of normal mice **(VII-IX):** mice were administered **Vilda** (20 mg/kg), **DDS** (50 mg/kg), and **Vilda/DDS** (Vilda (20 mg/kg) + DDS (50 mg/kg)), respectively. All treatments were orally administered for 14 consecutive days, starting from the sixth week post-infection (PI), except PZQ which was administered for two consecutive days at the seventh week PI. Vilda was freely dissolved in water, whereas, DDS and PZQ were freshly suspended in 2% Cremophor EL (Sigma-Aldrich, USA).

Two weeks after the end of treatment, mice were sacrificed by rapid decapitation without anesthesia. Blood samples were collected and centrifuged for 15 min at 3,000 g and then stored at -80 °C. After the liver perfusion process, liver tissues were immediately excised, weighed, and separated into various parts. The first part was homogenized and was kept frozen at -80 °C for subsequent analysis of the hepatic content of oxidative, inflammatory, apoptotic, and fibrotic markers. The second part was kept in 10% formalin for histopathological and immunohistochemical examinations and the last part of liver tissues along with the middle part of the small intestine were used for determining the number of eggs per gram of liver or intestinal tissues according to Cheever^66^.

### Parasitological analysis

Hepatic and portomesenteric vessels of mice were perfused according to Duvall et al.^67^ for worm recovery and subsequent counting. The percentage reduction of worm/egg burden in each treated group was calculated according to the following equation: % reduction = [(No. of worms/eggs in the control group) − (No. of worms/eggs in treated group)] / (No. of worms/eggs in the control group) × 100. The oogram pattern of the different egg developmental stages in small intestinal tissues was also examined according to Pellegrino et al.^43^, in which eggs at different stages of maturity were identified (I-IV) based on the embryo size and counted. Mature eggs containing fully developed miracidium and dead eggs (granular, dark, and semitransparent) were also counted in 3 fragments of the small intestine and the mean number of each stage was calculated.

### Hepatotoxicity markers

Hepatotoxicity biomarkers such as Alanine transaminase (ALT), Aspartate transaminase (AST), and albumin were determined using commercial kits (Biodiagnostics, Egypt) according to the manufacturer’s instructions. The liver index was calculated using the formula: liver weight/bodyweight × 100.

### Oxidative stress markers

Oxidative status in liver tissues was evaluated by measurement of superoxide dismutase (SOD) level and malondialdehyde (MDA) in liver homogenate using commercial kits (Biodiagnostics, Egypt) according to manufacturer’s instructions. While, the western blot technique was used for the assessment of both nuclear factor erythroid 2–related factor 2 (Nrf2) (Cat#sc-365949) and heme oxygenase-1 (HO-1) (Cat#Zsc-136960), (Santa Cruz Biotechnology, USA).

### Inflammatory and apoptotic markers

ELISA kits were used for the assessment of interleukin-1β (IL-1β) (Cat#E-EL-R0012) and tumor necrosis factor-alpha (TNF-α) (Cat#E-EL-R0019), and caspase-3 (Cat#E-EL-R0160) (Elabscience, USA) in liver homogenates according to manufacturer’s instructions. While, the western blot technique was used for the assessment of nuclear factor kappa-B p65 (NF-κB p65) (Cat#sc-8008, Santa Cruz Biotechnology, USA) and toll-like receptors 4 (TLR4) (Cat#sc-10741, Santa Cruz Biotechnology, USA), NOD-like receptor proteins (NLRP3) (Cat#EPR23094-1, Abcam, USA), and Caspase-1 (Cat#NBP1-45,433, Novus Biologicals, USA).

### Western blot analyses

Liver tissue homogenate was prepared using the ReadyPrep™ protein extraction kit (Catalog #163–2086) provided by Bio-Rad Inc, USA, to extract total protein samples and Bradford Protein Assay Kit (SK3041) for quantitative protein analysis was provided by Bio basic Inc (Markham Ontario L3R 8T4 Canada). A Bradford assay was performed according to manufacture instructions to determine protein concentration in each sample. Twenty μg of each sample protein was loaded with an equivalent volume of 2 × Laemmli sample buffer (4% SDS, 10% 2-mercaptoethanol, 20% glycerol, 0.004% bromophenol blue, and 0.125 M Tris HCl). Each previous mixture was boiled at 95 ^◦^ C for 5 min, PH at 6.8 to ensure denaturation of protein. Polyacrylamide gels were prepared using TGX Stain-Free™ and FastCast™ Acrylamide Kit (SDS-PAGE) (Cat#161–0181) which was provided by Bio-Rad Laboratories Inc. The membrane was blocked in tris-buffered saline with Tween 20 (TBST) buffer and 3% bovine serum albumin (BSA) at room temperature for 1 h. The primary antibodies of TLR4, NLRP3, NF-κB p65, HO-1, and Nrf2 were diluted as (1:1000) while, Caspase-1 as (1:500), in TBST according to manufactured instructions. In each primary antibody solution, incubation was done overnight at 4 °C against the blotted target protein. The blot was rinsed 3–5 times for 5 min with TBST. After that, incubation was done in the HRP-conjugated secondary antibody (Goat anti-rabbit IgG- HRP-1mg Goat mab -Novus Biologicals) solution against the blotted target protein for 1 h at room temperature. The blot was rinsed 3–5 times for 5 min with TBST. The chemiluminescent substrate Clarity TM Western ECL substrate (Cat#170–5060) were purchased from Bio-Rad Laboratories Inc (USA), was applied to the blot according to the manufacturer’s recommendation. The chemiluminescent signals were captured by a CCD camera-based imager and the band intensity of the target proteins against control sample beta-actin (housekeeping protein) was visualized using the ChemiDoc MP imager (Image J analysis software).

### Histopathological examinations

Liver tissues were sliced to 3–4 mm thick, fixed in 10% neutral buffered formalin (10% NBF), dehydrated in graded concentrations of ethanol, cleared in xylene, and embedded in paraffin. The paraffin blocks were sectioned with a microtome at 4–6 μm thickness and dyed with hematoxylin and eosin (H&E) stain to study general tissue structure (granuloma size (area), state of *S. mansoni* egg and granuloma type). Moreover, Ishak scoring system was applied for assessment of liver tissue as no fibrosis (0), Fibrous expansion of some portal areas, with or without short fibrous septa (1), Fibrous expansion of most portal areas, with or without short fibrous septa (2), Fibrous expansion of most portal areas with occasional portal to portal bridging (3), Fibrous expansion of portal areas with marked bridging (portal to portal as well as portal to central) (4), Marked bridging (portal–portal and/or portal–central) with occasional nodules (incomplete cirrhosis) (5), Cirrhosis, probable or definite (6) according to Goodman^68^.

For direct visualization of collagen fibers and histological changes assessment, staining was performed using Masson trichrome staining kit, (Sigma-Aldrich, St Louis, MO, USA)^69^. For conformation, Sirius Red/Fast Green stain was also applied (Sirius red stain (Sigma-Aldrich under the name of "Direct Red 80", Cat# 36–554-8–6, St Louis, MO, USA) and Fast green stain (Sigma-Aldrich, Fast green FCF (C.I. 42,053), CAS 2353–45-9, St Louis, MO, USA).

Collagen fibers in the former stain are marked with red color, whereas the non-collagen proteins look green in color^70^. Quantitative evaluation of H&E-stained sections and collagen fibers were measured 6 figures/ group, 40 × magnification lens.

### Immunohistochemical and fibrotic markers

ELISA kits were used for the assessment of matrix metallopeptidase-9 (MMP-9) (Cat#E-EL-R3021) (Elabscience, USA) in liver homogenates according to the manufacturer’s instructions.

Regarding immunohistochemical examination, thick deparaffinized liver Sects. (3–5 µm) were evaluated for the immunohistochemical reactivity of Anti-Rabbit α-SMA polyclonal Antibody (Cat#E-AB-34268, Elabscience, USA) and Anti-TGF-beta-1 polyclonal Antibody (Cat#ARG56429, Ari Gobio), following the manufacturer’s procedure. Slides were treated with 3% hydrogen peroxide, washed in PBS, and blocked in 1% bovine serum albumin. Afterward, they were incubated for an hour with primary antibody Anti-Rabbit α-SMA polyclonal Antibody (dilution: 1:50) and Anti-TGF beta 1 polyclonal Antibody (dilution: 1:50), then the slides were rinsed out by PBS and directly incubated for 20 min with a secondary antibody Horse Radish peroxidase Envision Kit and then washed out and incubated for 15 min with diaminobenzidine (DAB) (Sigma-Aldrich, USA) to detect antigen–antibody complex. After the slides were washed with PBS, counter-stained with hematoxylin, rehydrated, and cleared in xylene, they displayed positive brown immunostaining. Normal controls were included using non-immune serum in place of the primary or secondary antibodies. In IHC scoring, six fields (× 20) exhibit positive brown immunostaining were selected for evaluation in each serial section of the studied groups. Area % was determined for immune-stained sections via Leica QWin 500 image analyzer computer system (England) ‘L.A.S. software v.4’ (Leica Microsystems, Cambridge, UK). This image analyzer entails of Leica microscope (CH9435 Hee56rbrugg) (Leica Microsystems, Switzerland), a colored video camera, colored monitor and a hard disc of a Leica IBM personal computer linked to the microscope and managed by Leica QWin 500 software.

### Statistical analysis

Values are presented as Mean ± standard error of the mean (SEM). Data were examined for normality via the Kolmogorov–Smirnov test of normality. The outcomes of this test pointed out that most data were normally distributed (parametric data). Accordingly, descriptive analysis, One-Way ANOVA, and post hoc tests were operated for intergroup relationships using GraphPad Prism software version 9. Differences between means were considered statistically significant at P < 0.05.

## Supplementary Information

Supplementary Information.

## Data Availability

The datasets generated during the current study are available from the corresponding author upon reasonable request.

## Abbreviations

ALT: Alanine transaminase
AST: Aspartate transaminase
DDS: Diaminodiphenyl sulfone
DPPI-4: Dipeptidyl Peptidase IV inhibitor
H&E: Hematoxylin and eosin
HO-1: Heme Oxygenase-1
IL-1β: Interleukin-1β
MDA: Malondialdehyde
MMP-9: Matrix metallopeptidase-9
NF-κB p65: Nuclear factor kappa-B p65
NLRP3: NOD-like receptor proteins
Nrf2: The nuclear factor erythroid 2–related factor 2
*S. mansoni*: *Schistosoma mansoni*
SOD: Superoxide dismutase
TBST: Tris-buffered saline with Tween 20
TGF-β1: Transforming growth factor beta
TLR4: Toll-like receptors 4
TNF-α: Tumor necrosis factor-alpha
Vilda: Vildagliptin
α-SMA: Alpha Smooth Muscle Actin
