# Supplementary material for: The antifibrotic effect of Vildagliptin and Diaminodiphenyl Sulfone in murine schistosomiasis mansoni
Source: Sci Rep. 2025 Mar 24;15:10084. doi: 10.1038/s41598-025-91955-4 (PMC11933376; doi:10.1038/s41598-025-91955-4)
Supplement: Supplementary file 1 — Supplementary Information. [file 41598_2025_91955_MOESM1_ESM.pdf]

Full length gel of western blot

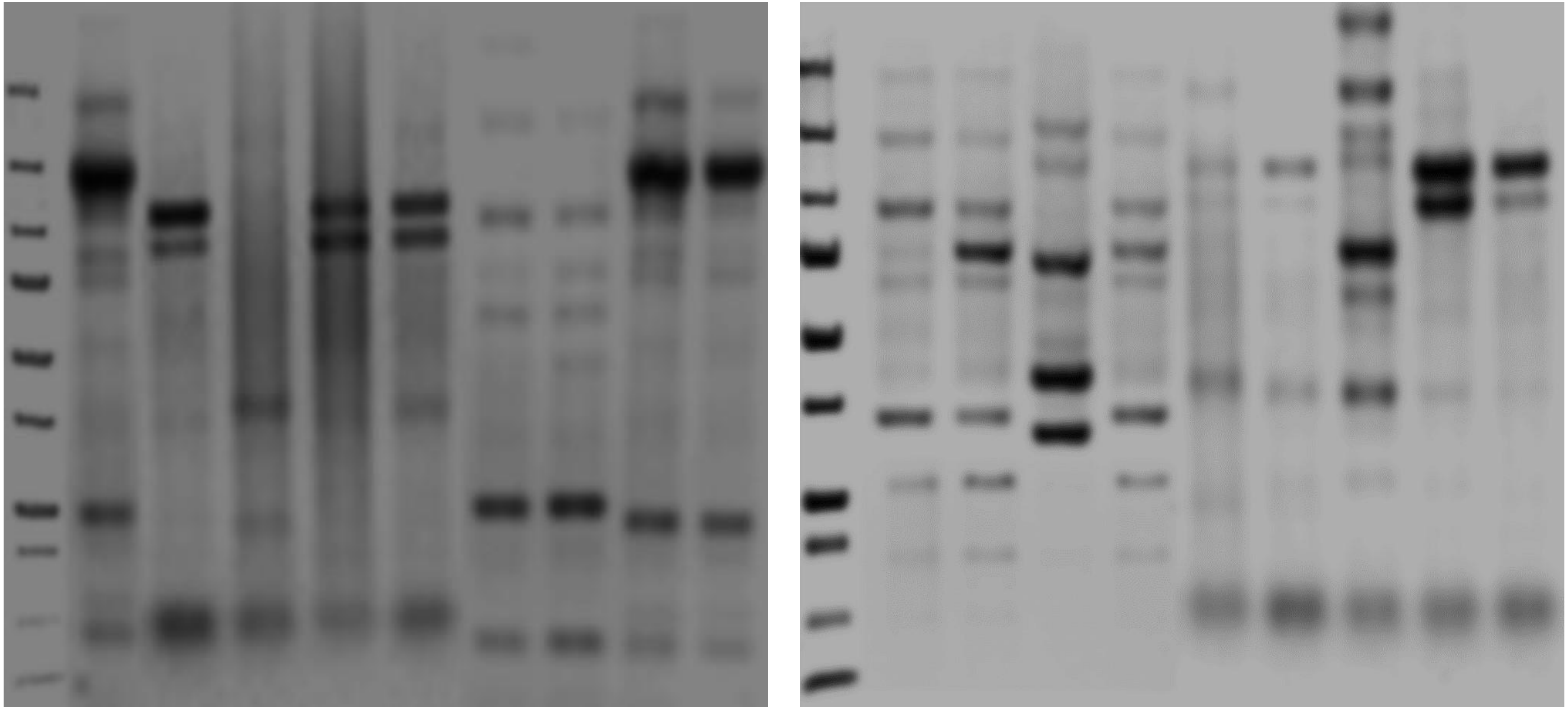

B- actin

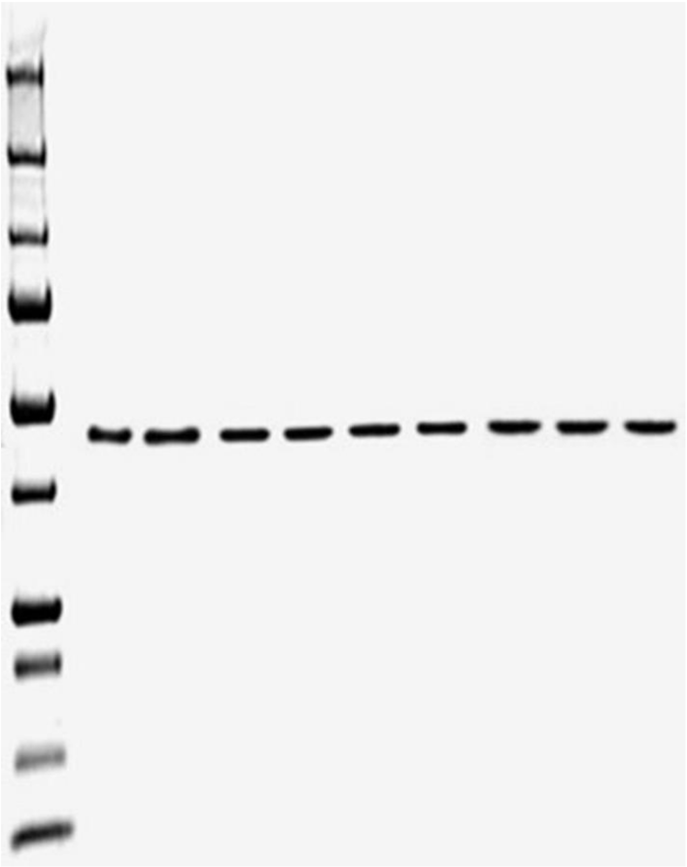

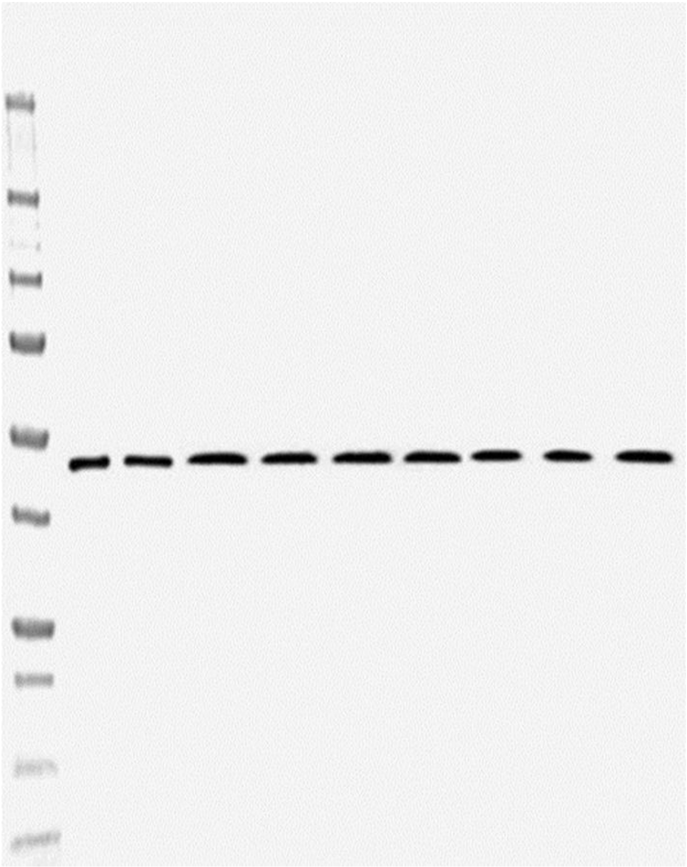

TLR4

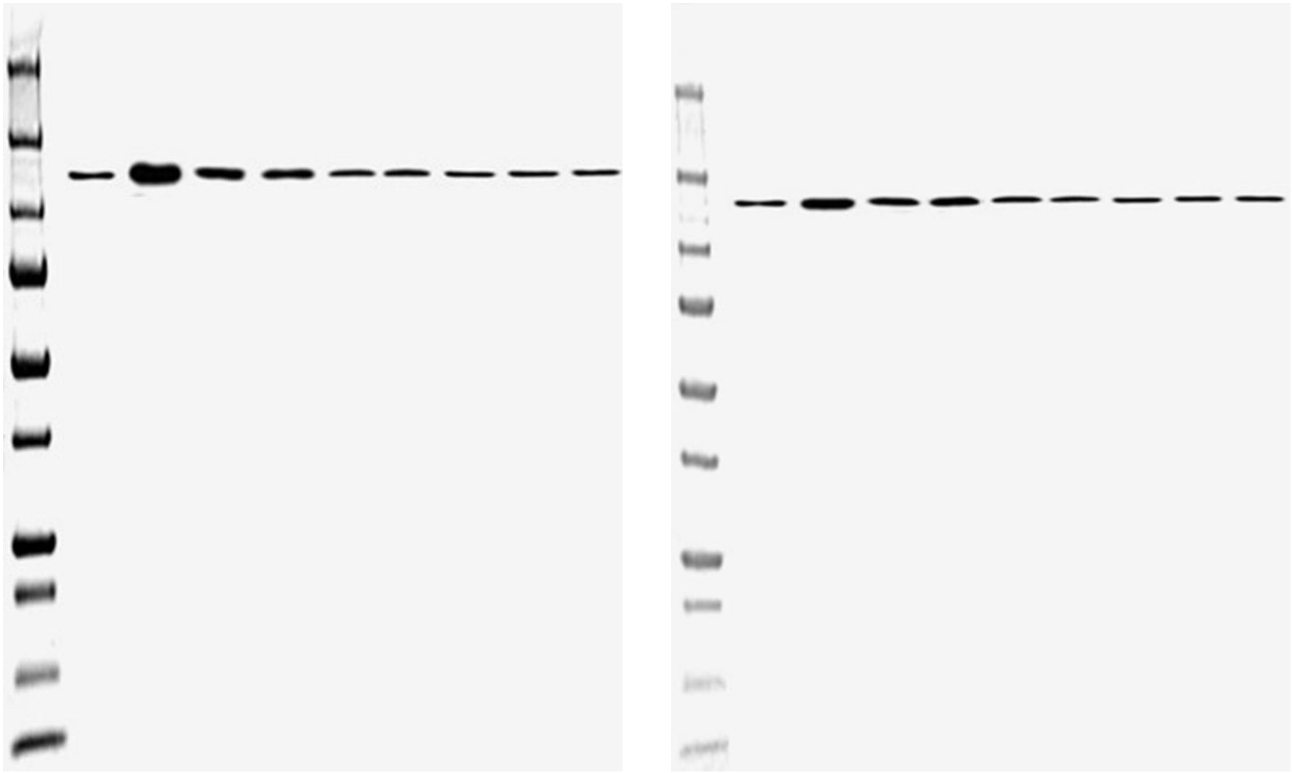

NLRP3

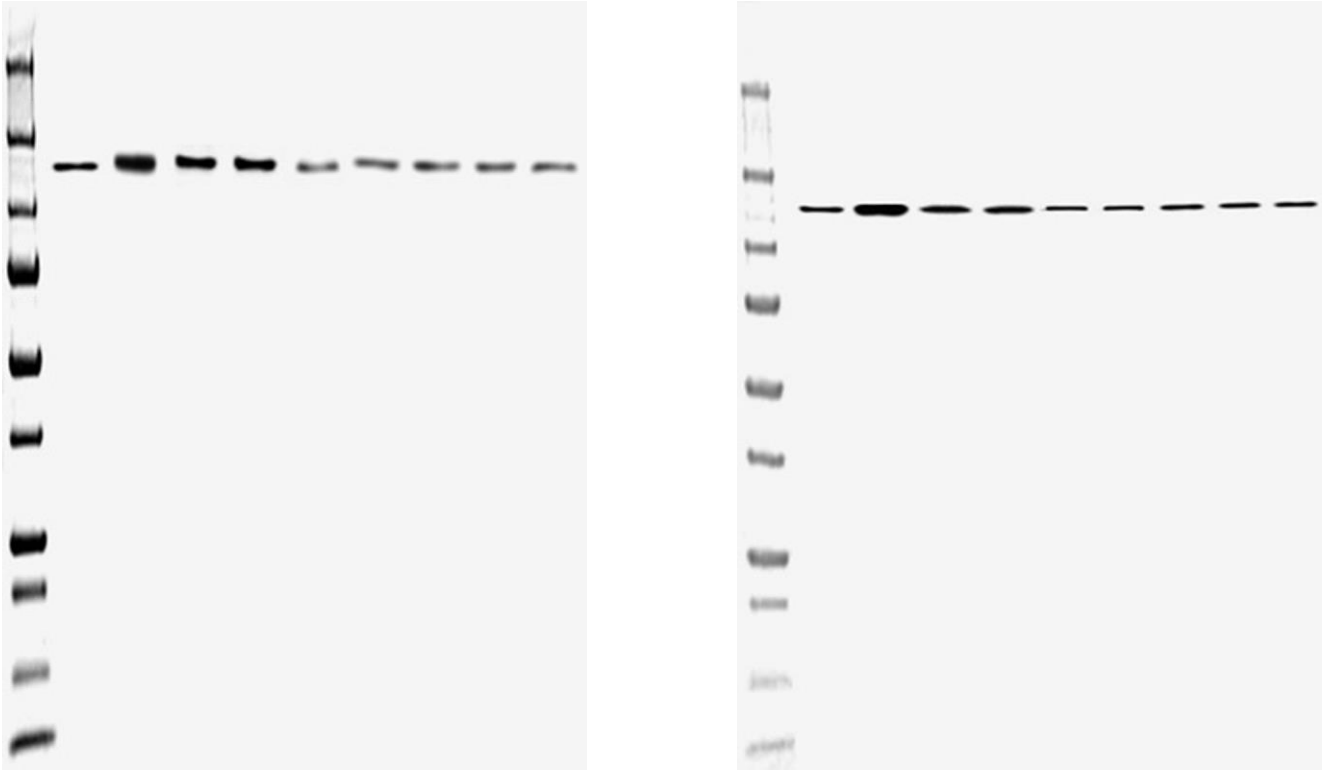

NFK-b p65

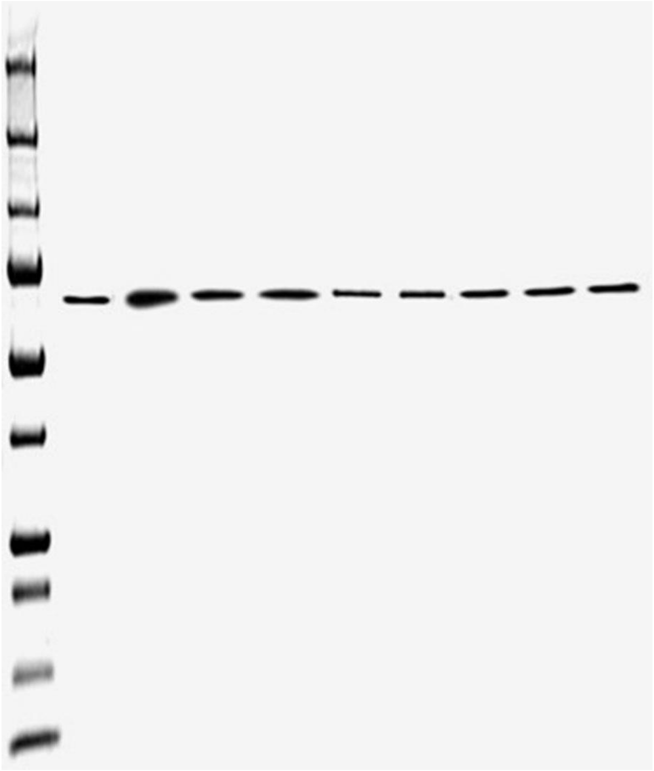
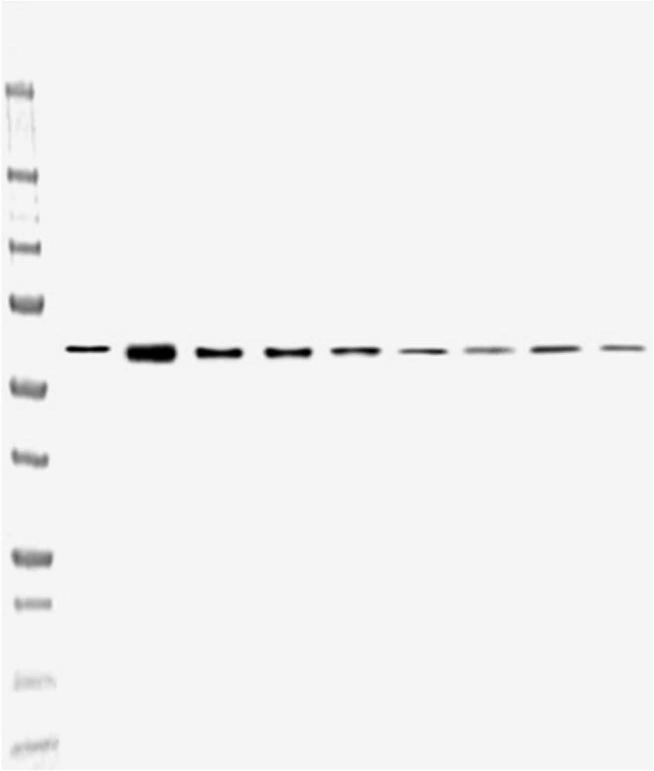

Caspase 1

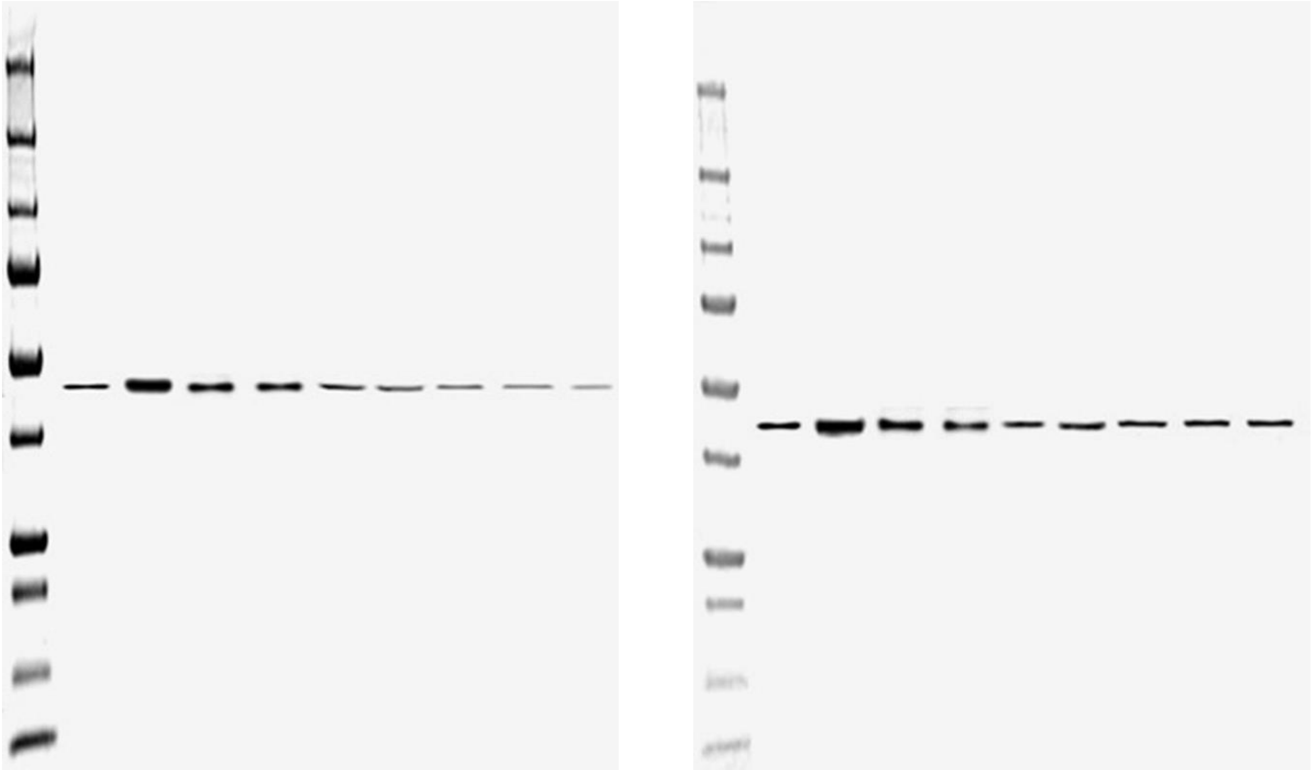

Nrf 2

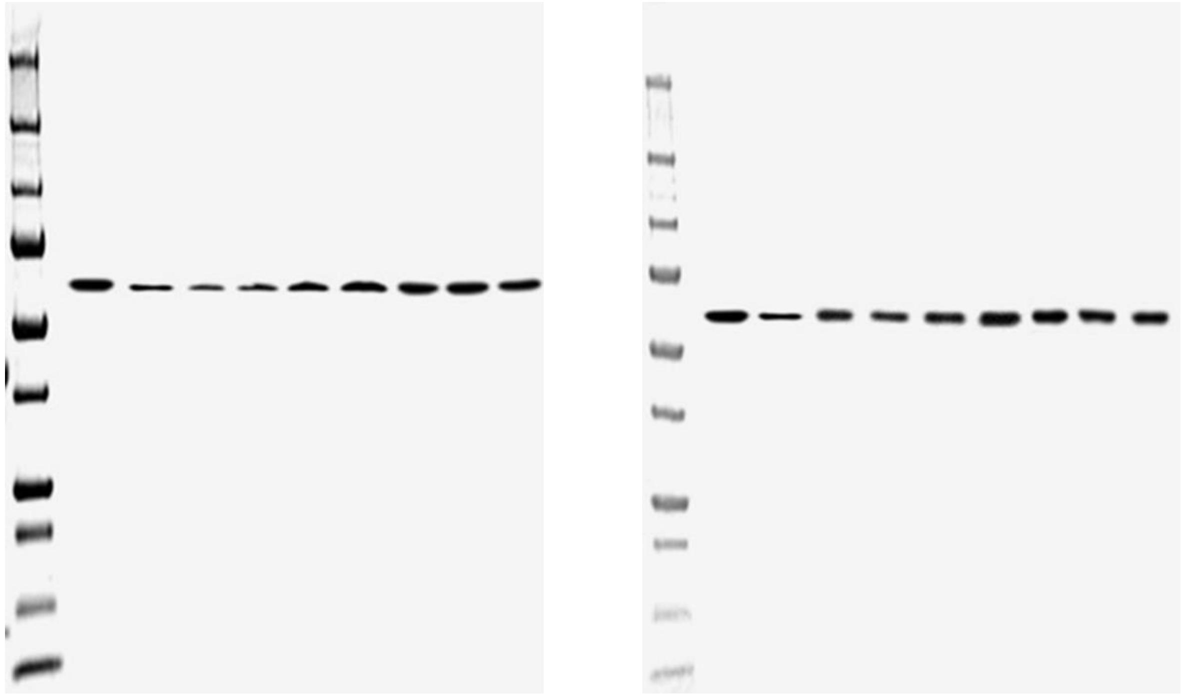

HO-1

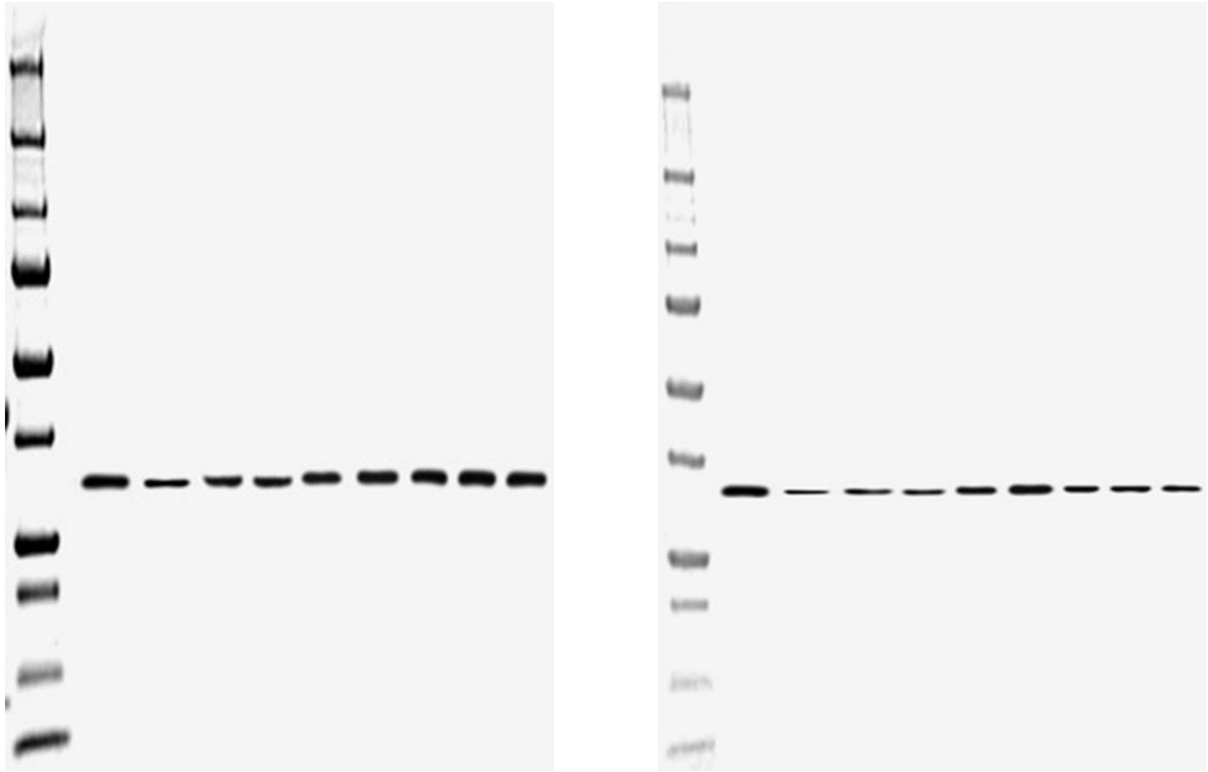

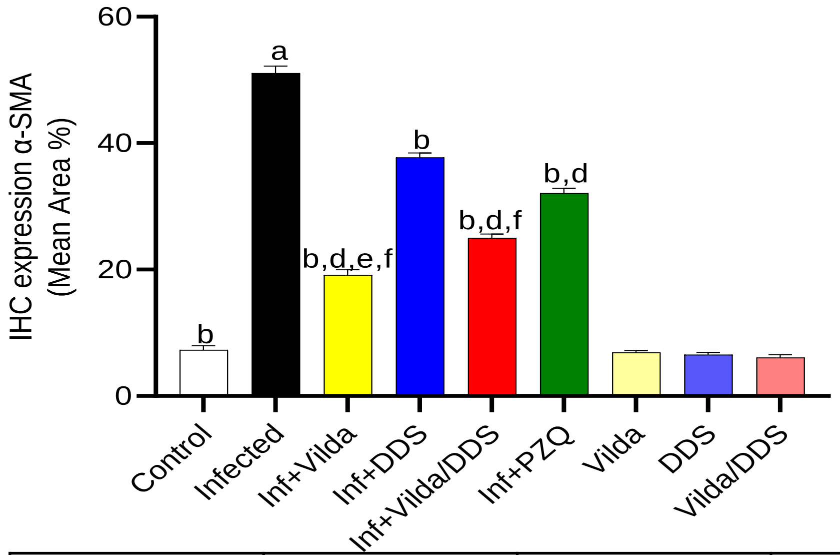

Prism Data of IHC of α-SMA

Control	Infected	Inf+Vilda	Inf+DDS	Inf+Vilda/DDS	Inf+PZQ	Vilda	DDS	Vilda/DDS
7.05	50.041	18.01	37.715	27.164	31.538	6.318	5.902	7.158
5.27	55.119	20.294	39.048	26.393	32.537	5.679	5.489	6.646
6.05	51.208	17.739	40.053	23.518	30.869	7.128	6.024	4.498
6.66	48.899	16.626	38.043	24.431	29.588	6.852	7.239	5.185
9.715	53.131	21.44	36.097	25.129	34.245	7.409	7.746	6.057
8.911	48.172	20.871	35.453	23.518	33.894	7.839	6.864	7.014

Prism Data of IHC of TGF-β

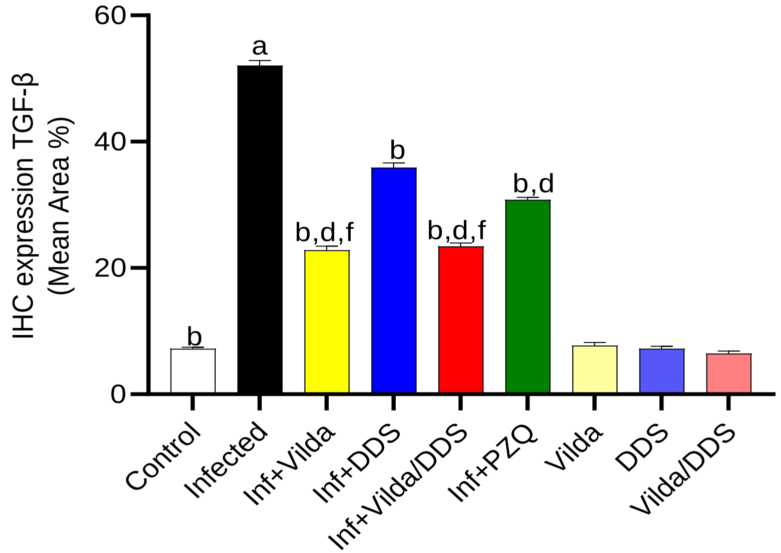

Control	Infected	Inf+Vilda	Inf+DDS	Inf+Vilda/DDS	Inf+PZQ	Vilda	DDS	Vilda/DDS
7.358	53.989	22.705	36.396	22.866	30.405	9.767	8.597	4.825
6.795	52.934	23.601	37.378	25.215	32.046	7.823	8.198	7.057
8.085	54.246	20.573	33.384	24.542	29.438	8.145	5.897	5.697
7.53	51.625	21.479	35.395	21.865	31.049	6.484	7.414	6.912
6.459	49.476	23.306	38.409	23.54	30.076	6.92	6.649	7.5
6.978	50.008	25.138	34.373	22.519	31.702	7.082	6.278	6.5
